# Experimental Study of YaJieShaBa Antialcoholic Hepatic Fibrosis Through TGF‐β1/Smad Signaling Pathway

**DOI:** 10.1155/mi/2319470

**Published:** 2026-06-12

**Authors:** Linao Zhang, Yuanmei Bai, Shifang Luo, Feifan Liu, Lijie Zheng, Yan Wan, Xue Wu, Qinghua Chen, Yuhuan Xie, Peixin Guo

**Affiliations:** ^1^ College of Chinese Medicine, Yunnan University of Chinese Medicine, Kunming, Yunnan, China, ynutcm.edu.cn; ^2^ Yunnan Key Laboratory of Dai and Yi Medicines, Yunnan University of Chinese Medicine, Kunming, Yunnan, China, ynutcm.edu.cn; ^3^ State Key Laboratory of Biotherapy, West China Hospital, Sichuan University, Chengdu, Sichuan, China, scu.edu.cn; ^4^ College of Ethnic Medicine, Yunnan University of Chinese Medicine, Kunming, Yunnan, China, ynutcm.edu.cn; ^5^ College of Basic Medical Sciences, Yunnan University of Chinese Medicine, Kunming, Yunnan, China, ynutcm.edu.cn; ^6^ School of Medicine, Yunnan College of Business Management, Kunming, Yunnan, China, ynjgy.com

**Keywords:** alcoholic hepatic fibrosis, mechanisms, pathway-focused qPCR array, pharmacodynamics, TGF-β1/Smad pathway

## Abstract

**Objective:**

This study aimed to clarify the pharmacodynamic effects of YaJieShaBa (YJSB) against alcoholic hepatic fibrosis (HF) and elucidate its mechanism in regulating the transforming growth factor‐β1 (TGF‐β1)/Smad pathway.

**Methods:**

Induce the alcoholic HF model in rats using 56% ethanol (10 mL/kg). The pharmacological efficacy of YJSB in combating liver fibrosis was evaluated through comprehensive assessments of key indicators: body weight, liver mass and index, biochemical liver function parameters (aspartate aminotransferase [AST] and alanine aminotransferase [ALT]), liver fibrosis biomarkers (type Ⅲ procollagen amino‐terminal propeptide [PⅢNP], type‐Ⅳ collagen (COL‐Ⅳ), laminin (LN), and hyaluronic acid [HA]), serum hydroxyproline (Hyp) and TGF‐β1 levels, hepatocyte homogenate levels of COL‐I, COL‐Ⅲ, and α‐smooth muscle actin (α‐SMA), along with histopathological changes observed in liver tissue via hematoxylin and eosin (H&E) staining, Ag staining, and Masson staining. Pathway‐focused qPCR array analysis was used to detect the expression of 72 genes related to signaling pathways such as TGF‐β1, Keap1‐Nrf2, and TLR4/MyD88 in liver tissue from the control group, model group, and YJSB group, identifying differentially expressed genes (DEGs) and key signaling pathways between the model group and the YJSB treatment group. Finally, based on the results of the pathway‐focused qPCR array, the mechanism of action of YJSB against HF was validated using ELISA, WB, and immunofluorescence methods. Concurrently, TGF‐β1 receptor inhibitors were employed in vitro experiments to determine whether YJSB could still provide additional protective effects when the TGF‐β1/Smad pathway was maximally blocked. Furthermore, after confirming that YJSB could inhibit TGF‐β1‐induced activation, a rescue experiment was conducted by adding exogenous TGF‐β1 to observe whether it could reverse the inhibitory effects of YJSB.

**Results:**

YJSB administration significantly increased body mass and decreased liver index in alcoholic HF rats. Serum levels of AST, ALT, PⅢNP, COL‐Ⅳ, LN, HA, Hyp, and TGF‐β1 were significantly reduced, as were the levels of COL‐I, Ⅲ, and α‐SMA in liver homogenates. Histological analyses, including H&E, Ag, and Masson staining, revealed a significant reduction in liver damage. Pathway‐focused qPCR array results showed that, compared with the blank group, 65 genes were upregulated and seven genes were downregulated in the model group, among which the relative expression levels of 40 genes were statistically significant (expression change factor ≥ 1 and *p*  < 0.05). Compared with the model group, 68 genes were downregulated, and four genes were upregulated in the YJSB group, with 34 genes showing statistically significant relative expression levels (fold change [FC] ≥ 2 and *p*  < 0.05). The DEGs were primarily enriched in the TGF‐β1 signaling pathway. Additionally, YJSB reduced the levels of inflammatory factors IL‐1β, TNF‐α, IL‐6, and IL‐8 in liver tissue homogenates while increasing SOD, GSH‐Px, and catalase (CAT) levels and decreasing MDA and ROS levels. Western blotting results showed that YJSB downregulated the expression levels of TGF‐β1, Smad2, Smad3, P‐Smad2, and P‐Smad3 in the liver. Immunofluorescence results indicated that YJSB downregulated the expression levels of Smad4 in hepatocyte nuclei. In vitro experiments demonstrated that the mechanism of YJSB involves primarily inhibiting TGF‐βR1 receptor activation, effectively downregulating P‐Smad2/3 protein levels, as validated by the TGF‐βR1 inhibitor LY2157299. Concurrently, in rescue experiments, the inhibitory effect of YJSB on P‐Smad2/3 protein was partially reversed by exogenous TGF‐β1, indicating that YJSB’s antifibrotic action is highly correlated with the TGF‐β1/Smad pathway.

**Conclusion:**

YJSB effectively inhibits the inflammatory and oxidative stress–related cascade by regulating the TGF‐β1/Smad signaling pathway (TSSP), thus suppressing the progression of alcoholic HF. This demonstrates that YJSB possesses potential for combating alcoholic HF in animal models, providing experimental evidence for its subsequent research and clinical application.

**Summary**

• The pharmacodynamic effects of YaJieShaBa (YJSB) on alcoholic hepatic fibrosis (HF) in a rat model were elucidated.
• The underlying mechanisms involved reducing the release of inflammatory and oxidative stress–related factors by regulating the transforming growth factor‐β1 (TGF‐β1)/Smad pathway, thereby suppressing the progression of alcoholic HF.
• This study established an experimental foundation for the clinical application of YJSB and the development of novel drugs to treat alcoholic HF.

## 1. Introduction

Hepatic fibrosis (HF) is a complex, fibrotic, and inflammatory process resulting from chronic liver injury and serves as the precursor to cirrhosis [1, 2]. Affecting 1%–2% of the global population, HF is responsible for over a million deaths annually, significantly impacting the quality of life and increasing financial burdens [3]. The progression of HF is associated with various underlying conditions, including alcohol‐related and nonalcoholic fatty liver disease, cholestatic disorders, drug‐induced hepatotoxicity, chronic viral hepatitis (hepatitis B and hepatitis C), and autoimmune hepatitis [4].

Several pharmacologic agents are currently used to manage HF, including colchicine (COL), adrenocorticotropic hormone (ACTH), and maroteric acid. COL functions by inhibiting the secretion of procollagen molecules [5], while ACTH suppresses collagen biosynthesis and reduces its deposition [6]. Despite these mechanisms, their clinical utility is limited by adverse effects [7]. Prolonged ACTH therapy may lead to sodium and water retention, increasing the risk of infections [6]. COL is associated with bone marrow suppression, severe gastrointestinal symptoms such as nausea and abdominal pain, and leukopenia. Thus, developing effective and safe drugs for preventing and treating HF is of great significance. Ethnomedicines, particularly natural medicines, have demonstrated unique advantages in combating HF [8, 9].

Dai medicine, practiced by the Chinese Dai people, is a traditional system with a systematic medical theory and rich clinical experience, exhibiting distinct national and local characteristics. It is a vital component of Chinese traditional medicine [9]. In Dai medicine, HF falls under the category of liver diseases caused by poisonous evil actions leading to dysfunction [8]. YaJieShaBa (YJSB) is a common formula used by Dai practitioners for liver diseases. YJSB has been used in Dai medicine to treat liver injury, hepatitis, and cirrhosis caused by drug, poison, or alcohol poisoning, demonstrating superior clinical efficacy [10]. In a clinical study involving 50 patients with hepatitis, 36 were cured, and 14 attained improvement, yielding an overall efficiency of 100% [9]. According to the prescription records (Table 1) [10], YJSB is composed of multiple botanical drugs in a fixed weight ratio of 14:5:7:5:6:18:15:20. Phytochemical studies have identified alkaloids, flavonoids, and triterpenoids as its major classes of constituents [11]. Alkaloids such as jatrorrhizine (C_20_H_20_NO_4_) and berberine (C_20_H_18_NO_4_^+^) exhibit anti‐inflammatory and antioxidant effects. Glycyrrhizic acid (C_42_H_62_O_16_), a triterpenoid, possesses anti‐inflammatory and antiviral properties [10].

**Table 1:** Contents of YJSB decoction. All botanical drugs were obtained from Xishuangbanna Dai Nationality Hospital in Yunnan Province, China.

Local name	Species name	Family name	Drug name
Weng shang hai	*Arundina graminifolia* (D. Don) Hochr	Orchidaceae	Arundinae herba
Dai bai jie	*Dregea sinensis* Hemsl.	Apocynaceae	Dregeae radix
Hei tao han	*Fibraurea recisa* Pierre.	Menispermaceae	Fibraureae caulis
He bie	*Pueraria montana var. Lobata* (Willd.) Maesen & S.M.Almeida ex Sanjappa & Predeep.	Fabaceae	Puerariae lobatae radix
Deng hei han	*Mappianthus iodoides* Hand. ‐Mazz.	Icacinaceae	Mappianthuse caulis
Jie long meng la	*Anodendron nervosum* Kerr.	Apocynaceae	Anodendrone caulis
Bai hua chou mu dan gen	*Clerodendrum chinense* (Osbeck) Mabb.	Lamiaceae	Clerodendrume radix
Sha yin	*Glycyrrhiza uralensis* Fisch. ex DC.	Fabaceae	Glycyrrhizae radix et rhizoma

Oxidative stress activates multiple transcription factors, mediates crucial signaling pathways, regulates protein expression, and stimulates hepatic stellate cells (HSCs), leading to inflammatory response, fibrosis, and apoptosis [10, 12]. It plays a critical role in the pathogenesis of HF by activating TGF‐β and mediating downstream signaling pathways [13, 14]. Transforming growth factor‐β1 (TGF‐β1), the cytokine most closely associated with HF progression, regulates the synthesis and decomposition of the extracellular matrix (ECM), induces cell differentiation, and modulates immunity [15, 16]. Upon stimulation by various injuries, HSCs enhance TGF‐β1 levels [17], which bind to cell membrane receptors and activate the TGF‐β1/Smad signaling pathway (TSSP). This activation forms a heterodimeric complex that further triggers the phosphorylation of Smad2 and Smad3 [18, 19]. The complex then dissociates from the cell membrane, enters the cytoplasm, forms a multimer with Smad4, translocates to the nucleus, and activates the transcription of target genes, thereby leading to collagen production and HF [20].

A previous study by our group demonstrated that YJSB exerts a protective effect against both CCl_4_‐induced HF in rats and bile duct ligation–induced HF in mice, accompanied by a significant reduction in TGF‐β1 levels [10, 19]. TGF‐β1 is widely recognized as a key regulator of liver fibrosis, driving the activation and phenotypic transformation of HSCs as well as excessive ECM deposition [21, 22]. Therefore, it was hypothesized that YJSB’s antialcoholic HF effects might involve regulating the TSSP, reducing TGF‐β1 overexpression, promoting ECM degradation, and enhancing hepatoprotective effects.

Pathway‐focused qPCR array integrates DNA microarray technology with the analysis of specific biological pathways to effectively eliminate gene information unrelated to the research objective in traditional expression profile chips, thereby precisely focusing on genes highly correlated with specific diseases [23]. This technology provides a targeted detection platform for efficiently identifying differentially expressed genes (DEGs) involved in various biological processes and has become an important research method for elucidating disease pathogenesis or drug mechanisms of action [24].

This study employed pathway‐focused qPCR array technology to analyze DEGs in rat livers across groups, providing direction for elucidating the potential mechanisms underlying YJSB treatment of alcoholic HF. Using an animal model of alcoholic HF and a TGF‐β1–treated HSC‐T6 cell model, we investigated whether YJSB exerts therapeutic effects on alcoholic HF by modulating the TSSP to suppress the release of inflammation‐ and oxidative stress–related factors.

## 2. Materials

### 2.1. Drugs

The YJSB formula consists of eight botanical drugs combined in a fixed weight ratio of 14:5:7:5:618:15:20 (Table 1). Authentication of these crude drugs was performed by Prof. Yanfang Lin, a renowned Dai medicine specialist. COL (batch number 20210702) was purchased from Yunnan Botanical Pharmaceutical Co., Ltd. (Yunnan, China); it is known to effectively reduce the severity of alcohol‐induced HF [25]. Compound turtle shell soft liver tablets (CBRTs, batch number C0121093) were obtained from Neimenggu Furui Medical Technology Co., Ltd. (Neimeng, China); this agent suppresses adipocyte proliferation, reduces collagen synthesis, and effectively blocks early‐stage fibrotic progression [26]. COL and CBRT were used as positive control drugs.

### 2.2. Reagents

Aspartate aminotransferase (AST), alanine aminotransferase (ALT), SOD, MDA, GSH‐Px, catalase (CAT), and ROS determination kits (batch numbers: 20230328, 20230329, 20230419, A003‐1‐2, A005‐1‐2, A008‐1‐2, and A006‐1‐3, respectively) were obtained from Nanjing Jiancheng Bioengineering Institute, Nanjing, China. Kits for PIIINP, type‐Ⅳ collagen (COL‐IV), laminin (LN), hyaluronic acid (HA), hydroxyproline (Hyp), TGF‐β1, TNF‐α, IL‐1β, IL‐6, and IL‐8 (Batch numbers: MB‐5750 A, MB‐5752 A, MB‐6617 A, MB‐3250 A, MB‐4513 A, MB‐5751 A, MB‐3841 A, MB‐5970 A, MB‐7090 A, and MB‐6614 A, respectively) were sourced from Jiangsu Enzymatic Labeling Bioscience Co., Ltd., Jiangsu, China. TGF‐βR1 inhibitor LY2157299 was purchased from AmBeed (Batch number A150887). TGF‐β1 was purchased from MCE (batch number HY‐P70648).

### 2.3. Experimental Animals

Seventy male Sprague–Dawley (SD) rats (180–220 g) were obtained from Sibeifu (Beijing) Biotechnology Co., Ltd. (Certificate of Conformity Number SCXK (Beijing) 2019‐0010). The rats were housed in an environment maintained at 23°C ± 2 with 50% ± 5 humidity, with free access to food and water. All experimental procedures were performed in accordance with the Guide for the Care and Use of Laboratory Animals and were approved by the Ethics Committee of Yunnan University of Traditional Chinese Medicine (Animal Ethics Number R‐062023033).

## 3. Methods

### 3.1. Drug Dosage

According to a previous study [10], the doses of YJSB used in this study were 1.1 g/kg, 2.2 g/kg, and 4.4 g/kg, with the 2.2 g/kg dose being close to the clinically common dose converted into a rat equivalent. During the experiment, no signs of poisoning were observed, and postexperimental organ examinations revealed no abnormal changes, demonstrating the safety of these doses. Based on the literature, the gavage doses for CBRT and COL were 0.54 g/kg [25] and 0.27 mg/kg [27], respectively.

### 3.2. Modeling, Grouping, and Drug Administration

**Figure 1:**
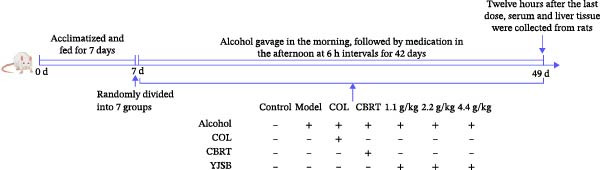
Timeline of a replicated rat model of HF.

Prior to the experiment, 70 healthy male SD rats were fed adaptively for 7 days. The animals were then randomly divided into five groups based on body weight: control group, model group, COL group, CBRT group, and YJSB group, with doses of 1.1, 2.2, and 4.4 g/kg, respectively, with 10 animals in each group. Except for the control group, all other groups were administered 56% alcohol by gavage in the morning. After a 6‐h interval, the animals in each group received the corresponding drug doses listed in Table 2 by gavage once daily for 42 consecutive days, with a gavage volume of 10 mL/kg (Figure 1). After the final administration of YJSB, the rats were deprived of water for 12 h and weighed. They were then anesthetized with 1% pentobarbital sodium (40 mg/kg) injected intraperitoneally. Blood was collected from the abdominal aorta, and then euthanasia was performed by administering an overdose of sodium pentobarbital (150 mg/kg). The blood was centrifuged at 4°C and 4000 rpm for 10 min to separate the serum, which was then stored at −80°C for future use.

**Table 2:** Doses administered to animals in each group.

Groups	Dose
Control	Equivalent distilled water
Model	Equivalent distilled water
COL	0.27 mg/kg
CBRT	0.54 g/kg
YJSB small dose	1.1 g/kg
YJSB medium dose	2.2 g/kg
YJSB large dose	4.4 g/kg

### 3.3. Observation of the Routine Status of the Rats

The rats were weighed weekly throughout the experiment. After the final modeling, observations of fur quality, mental state, morphology, and activity were recorded for a comprehensive evaluation of the rats’ statuses.

### 3.4. The Biochemical Indices of Liver Function

Take rat serum and determine the activity levels of AST and ALT using a colorimetric method according to the instructions in the kit manual.

### 3.5. Degree of HF

Serum levels of type Ⅲ procollagen amino‐terminal propeptide (PⅢNP), COL‐Ⅳ, LN, HA, Hyp, and TGF‐β1 were measured using ELISA.

### 3.6. Observation of Liver Morphology

Following euthanasia, the animals in each group were dissected to observe the appearance and morphology of the livers, noting characteristics such as color and texture.

### 3.7. Determination of Liver Mass and Index

The livers were rinsed, blotted on filter papers, and weighed. The right lobes were fixed in 4% neutral formaldehyde for subsequent pathological examinations, while the remaining tissue was preserved at −80 °C. The liver index was calculated as follows:

Liver index (%)=liver mass (g)body mass (g)×100%.

### 3.8. Determination of Collagen Deposition

For this experiment, 100 mg of liver tissue was added to 900 μL of saline, ground, and homogenized using a high‐frequency, high‐speed cryo‐mill. The homogenate was then centrifuged at 5000 rpm and 4°C for 15 min, and the supernatant was collected to measure the levels of α‐smooth muscle actin (α‐SMA), COL‐I, and COL‐Ⅲ using ELISA.

### 3.9. Hematoxylin and Eosin (H&E), Masson, and Ag Staining

The right hepatic lobe was subjected to H&E staining, Masson staining, and Ag staining to assess changes in hepatic inflammatory infiltration, collagen fibers, and reticular fibers among the groups.

### 3.10. RNA Extraction and cDNA Synthesis

Take 100 mg of liver tissue samples from the normal control group, model group, and YJSB treatment group, and extract total RNA using TRIzol reagent. Digest the RNA samples with DNase I to remove any genomic DNA (gDNA) contamination, ensuring the accuracy of subsequent gene expression detection results. Subsequently, mRNA was purified using the RNeasy MinElute Purification Kit (Qiagen). The concentration and purity of the mRNA were determined using a UV spectrophotometer, and the purified mRNA was characterized by agarose gel electrophoresis to verify RNA integrity and the absence of residual gDNA contaminants. Finally, an appropriate amount of purified mRNA samples that passed quality control were subjected to reverse transcription using reagents such as SuperScript III Reverse Transcriptase and RNase Inhibitor to synthesize cDNA.

### 3.11. Real‐Time PCR Amplification and Chip Scanning

The model group and the blank group, as well as the model group and the treatment group, were subjected to chip scanning according to the following procedure: Synthetic cDNA was mixed with Arraystar SYBR Green qPCR Master Mix (ROX+) (AS‐MR‐006‐5, Arraystar), added to a 384‐well plate, and subjected to real‐time PCR amplification on an ABI 7900 PCR instrument. In this 384‐well chip format, 72 individual wells simultaneously detect genes related to liver fibrosis. Additionally, three individual wells contain housekeeping genes (ACTB, B2M, and GAPDH) for chip data standardization. One well serves as a gDNA reference (GDC) to detect DNA contamination in the sample. Three replicate wells serve as reverse transcription references (RNA Spike‐in) to assess the efficiency of the RT reaction. Additionally, two replicate wells are designated as positive PCR references (PPC) to reflect PCR reaction efficiency. These wells include artificially synthesized DNA sequences and corresponding primer pairs for the reaction.

### 3.12. Detection Data Analysis

Data analysis employed the ΔΔCT method corrected by housekeeping genes: The average CT values of the three housekeeping genes were used as internal references to normalize chip data [28]. After calculating the average CT values of genes in the blank group, model group, and treatment group, the intergroup expression difference multiples were calculated using the 2^−ΔΔCT^ formula [29]. SPSS 20.0 was used for unpaired *t*‐tests. Different genes were defined as those meeting the following criteria: |fold change [FC]| ≥ 2 and *p*  < 0.05 [30].

### 3.13. Determination of Inflammation‐Related Factors

The contents of TNF‐α, IL‐1β, IL‐6, and IL‐8 in the liver homogenates were determined using ELISA.

### 3.14. Determination of Oxidative Stress–Related Factors

The content of MDA, SOD, GSH‐Px, CAT, and ROS in liver homogenates was determined using colorimetry.

### 3.15. Western Blotting Method to Determine Protein Expression

Homogenize liver tissue in RIPA lysis buffer (1:9, w/v), lyse on ice for 30 min, and then centrifuge at 4000 rpm at 4°C for 10 min. Collect the supernatant and quantify the protein using the BCA method. The supernatant was collected, and the protein concentration was measured using the BCA protein assay kit. The protein solution was mixed with 5× loading buffer at a 4:1 ratio to adjust the protein concentration to 2 mg/mL. Proteins were separated by SDS‐polyacrylamide gel electrophoresis and transferred to a PVDF membrane. The membrane was blocked with 5% nonfat milk for 1 h, followed by overnight incubation at 4°C with primary antibodies for anti‐TGF‐β1 (1:2000, ABclonal, A27715), anti‐Smad2 (1:1000, ABclonal, A16912), anti‐Smad3 (1:1000, ABclonal, A22133), anti‐P‐Smad2 (1:1000, ABclonal, AP0269), anti‐P‐Smad3 (1:1000, ABclonal, AP0554), and anti‐GAPDH (1:5000, ABclonal, A19056). The next day, the membrane was washed and incubated with rabbit antirat secondary antibody (1:10000, ABclonal, AS104) for 1 h. After washing, the membrane was imaged using a chemiluminescence imaging system. Data were analyzed using Bright Analysis Software.

### 3.16. Fluorescence Immunoassay of Smad4

The anti‐Smad4 (1:100, ABclonal, A5657) primary antibody was added to observe the dehydrated liver tissue sections. The secondary FITC‐antibody (1:400) reagent was used, and 4’,6‐diamidino‐2‐phenylindole (DAPI) was applied to restain cell nuclei. The sections were sealed and observed for Smad4 protein expression under a fluorescence microscope.

### 3.17. HSC‐T6 Cell Culture and Handling

Rat HSCs (HSC‐T6) were purchased from Boster Biological Technology (Wuhan, China) and cultured in DMEM medium supplemented with 10% fetal bovine serum (FBS) and 50 μg/mL penicillin/streptomycin at 37°C in a 5% CO_2_ incubator. A liver fibrosis cell model was established by treating HSC‐T6 cells with TGF‐β1 (10 ng/mL) for 48 h [15]. Based on the previous cytotoxicity study results from our research group, a 125 ng/mL solution of YJSB was selected for the in vitro study [19]. HSC‐T6 cells were divided into six groups: (1) normal control group; (2) TGF‐β1 group; (3) TGF‐β1 + LY2157299 (10 μM, treated for 8 h) group; (4) TGF‐β1 + YJSB (125 ng/mL, treated for 24 h) group; (5) TGF‐β1 + LY2157299 (10 μM) + YJSB (125 ng/mL) group; and (6) TGF‐β1 rescue group: cells treated with TGF‐β1 (10 ng/mL) for 48 h, followed by YJSB (125 ng/mL) for 24 h, and then high‐dose TGF‐β1 (20 ng/mL) for 24 h.

### 3.18. Effects of YJSB on the Expression of Smad2, P‐Smad2, Smad3, and P‐Smad3 Proteins in HSC‐T6 Cell Lysates (Western Blotting)

HSC‐T6 cells were seeded at 1 × 10^5^ cells/mL in culture dishes for HSC‐T6 grouping, modeling, and drug administration. Remove the culture medium and wash once with PBS. Add 1 mL of the following mixture to each dish: (RIPA lysis buffer: PMSF: phosphatase inhibitor = 100:1: 1) to each dish. Incubate for 30 min to lyse cells. Collect the lysate and determine the total protein concentration of each sample using the BCA method. Mix the protein solution with 5× loading buffer at a 4:1 ratio to standardize protein concentration. Proteins were separated by SDS‐polyacrylamide gel electrophoresis and transferred to a PVDF membrane. The membrane was blocked with 5% skim milk powder for 1 h, followed by overnight incubation at 4°C with anti‐Smad2 (1:1000, Shanghai Yamei, R013765), anti‐Smad3 (1:1000, Shanghai Yamei, R011766), anti‐P‐Smad2 (1:1000, Cell Signaling Technology, 18338T), anti‐P‐Smad3 (1:1000, Shanghai Yamei, R013553), and anti‐GAPDH (1:6000, Boster, BM3874) primary antibodies. The following day, after washing the membrane, add rabbit antigoat secondary antibody (1:10,000, ABclonal, AS104) and incubate for 1 h. After washing, image the membrane using a chemiluminescence imaging system. Data were analyzed using Bright Analysis Software.

### 3.19. Statistical Analyses

Statistical analysis was conducted using the SPSS 26.0 software. For normally distributed data, one‐way ANOVA was employed for pairwise comparisons among multiple groups. The LSD method was utilized for homogeneous variances, whereas the Dunnett’s T3 method was employed for heterogeneous variances. For non‐normally distributed data, nonparametric tests were utilized. Additionally, a *p*‐value of less than 0.05 indicated statistical significance.

## 4. Results

### 4.1. Quality and Composition of YJSB

YJSB has been prepared and utilized by Xishuangbanna Dai Hospital for many years [11]. The extraction of YJSB strictly followed the methodology established in previous studies, which largely ensured the stability and reproducibility of its pharmacological effects. Furthermore, previous work also conducted quality standard studies on YJSB and determined the content of its main components, berberine and palmatine [10].

### 4.2. YJSB Improves the General State of Alcoholic HF Rats

In this study, control group rats exhibited good physical activity, normal hair color, regular diet, and no adverse reactions. Conversely, rats in the model group displayed reduced physical activity, dull fur color, and mental fatigue. Improvements were observed in the YJSB‐treatment groups compared to the model group, with rats in the treatment groups showing good mental state, smooth hair color, normal diet, and regular feces.

### 4.3. YJSB Improves Liver Morphology in Alcoholic HF Rats

The physiological status and structural characteristics of the liver in the alcoholic HF rat model were assessed. Livers in the control group appeared normal, bright red, soft to the touch, and had smooth surfaces. In contrast, livers in the model group displayed several ascites, dark red color, uneven surfaces, hard texture, and large areas with white nodule–like changes. However, the treatment group livers showed a reduction in ascites, flat surfaces, soft texture, red color, and a significant reduction in white nodules.

### 4.4. YJSB Increased Body Weight and Liver Mass in Alcoholic HF Rats While Decreasing the Liver Index

**Figure 2:**
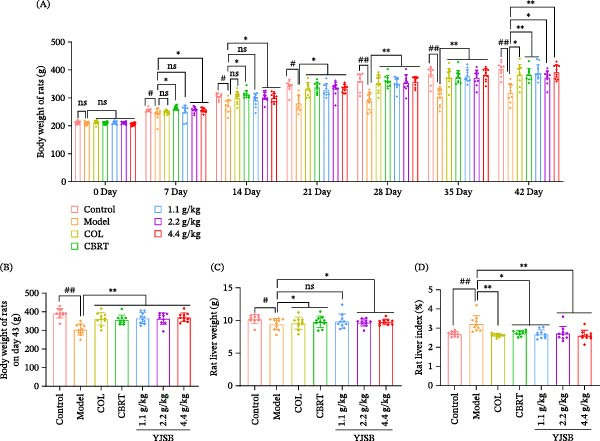
YJSB enhanced the body and liver masses of rats but decreased their liver index (*n* = 10). (A) Body mass of rats on days 0, 7, 14, 21, 28, 35, and 42. (B) Body mass of rats 24 h after the last administration on day 43. (C) Liver mass of rats 24 h after the last administration on day 43. (D) Liver index of rats 24 h after the last administration on day 43. Comparison with the control group: ^#^ *p* < 0.05 and ^##^ *p* < 0.01. Comparison with the model group:  ^∗^ *p* < 0.05 and  ^∗∗^ *p* < 0.01; ns: *p*  > 0.05.

The effects of YJSB on HF in rats were investigated by measuring body mass, liver mass, and liver index. The body mass of the model group rats was significantly reduced on the 7^th^, 14^th^, 21^st^, 28^th^, 35^th^, and 42^nd^ days compared to the control group (*p*  < 0.05 or < 0.01) (Figure 2A). The body weight of the 1.1, 2.2, and 4.4 g/kg YJSB‐treated rats was significantly higher than that of the model group on the 21^st^, 28^th^, 35^th^, and 42^nd^ days (*p*  < 0.05 or < 0.01) (Figure 2A). Additionally, the 2.2 and 4.4 g/kg YJSB‐treated groups exhibited a significant increase in body weight on the 7^th^ and 14^th^ days (*p*  < 0.05 or < 0.01) (Figure 2A), while the 1.1 g/kg YJSB‐treated group exhibited an increasing trend (*p*  > 0.05) (Figure 2A).

The last administration of YJSB was on the 43^rd^ day. After 24 h, the model group exhibited a significant reduction in body and liver masses, whereas the liver index increased compared to the control group (*p*  < 0.05 or < 0.01) (Figure 2B–D). In contrast, the 1.1, 2.2, and 4.4 g/kg YJSB‐treated groups exhibited a significant increase in body mass and a significant reduction in the liver index compared to the model group. The liver mass of the 1.1 (*p*  > 0.05), 2.2, and 4.4 g/kg YJSB‐treated groups was significantly elevated (*p*  < 0.05 or < 0.01) (Figure 2B–D).

### 4.5. Effect of YJSB on the Biochemical Indices of Liver Function

**Figure 3:**
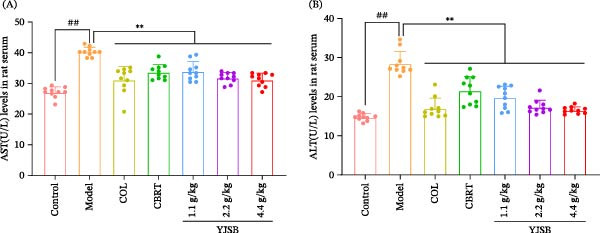
YJSB improved the liver function in alcoholic HF rats (*n* = 10). Serum levels of AST (A) and ALT (B). Comparison with the control group, ^##^ *p* < 0.01. Comparison with the model group,  ^∗∗^ *p* < 0.01.

The serum levels of ALT and AST were examined to assess alcohol‐induced hepatocellular injury. These levels were significantly elevated in the model group compared to the control group (*p* < 0.01) (Figure 3A,B). In contrast, ALT and AST levels in the 1.1, 2.2, and 4.4 g/kg YJSB‐treated groups were significantly lower than those in the model group (*p* < 0.01) (Figure 3A,B). These results indicate the positive effect of YJSB on liver function.

### 4.6. YJSB Reduced the Levels of the Four Biomarkers of Alcoholic HF (HA, PⅢNP, COL‐Ⅳ, and LN)

**Figure 4:**
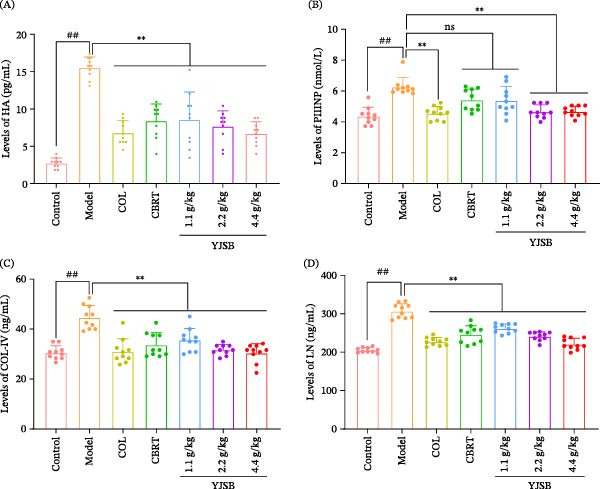
YJSB lowered the serum contents of HA, PⅢNP, COL‐Ⅳ, and LN in rats with alcoholic HF (*n* = 10). Serum levels of HA (A), PⅢNP (B), COL‐Ⅳ (C), and LN (D) in rats. Comparison with the control group, ^##^ *p* < 0.01. Comparison with the model group,  ^∗∗^ *p* < 0.01; ns: *p*  > 0.05.

The serum levels of HA, PⅢNP, COL‐Ⅳ, and LN were significantly higher in the model group compared to the control group (*p* < 0.01) (Figure 4A–D). Conversely, HA, COL‐Ⅳ, and LN levels were significantly lower in the 1.1, 2.2, and 4.4 g/kg YJSB‐treated groups compared to the model group (*p* < 0.01) (Figure 4A,C,D). Similarly, the serum PⅢNP level was significantly lower in the 2.2 and 4.4 g/kg YJSB‐treated groups compared to the model group (*p* < 0.01) (Figure 4B), with a nonsignificant reduction observed in the 1.1 g/kg YJSB‐treated group (*p* > 0.05) (Figure 4B). These findings indicate that YJSB can effectively reduce the production of HA, PⅢNP, COL‐Ⅳ, and LN in the liver and has a significant antialcoholic HF effect.

### 4.7. YJSB Diminished the Serum Levels of Hyp and TGF‐β1 in Rats With Alcoholic HF

**Figure 5:**
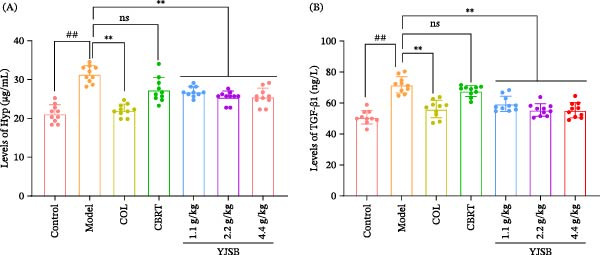
YJSB lowered the serum content of Hyp and TGF‐β1 in rats with alcoholic HF (*n* = 10). (A) Serum content of Hyp in rats. (B) Serum content of TGF‐β1 in rats. Comparison with the control group, ^##^ *p* < 0.01. Comparison with the model group,  ^∗∗^ *p* < 0.01; ns: *p*  > 0.05.

The experimental results indicate that the serum levels of Hyp and TGF‐β1 were significantly higher in the model group compared to the control group (*p* < 0.01) (Figure 5A,B). However, these levels were significantly reduced in the 1.1, 2.2, and 4.4 g/kg YJSB‐treated groups compared to the model group (*p* < 0.01) (Figure 5A,B). The results showed that YJSB treatment significantly downregulated the levels of Hyp and TGF‐β1 in the rat liver, effectively inhibiting ECM deposition and the progression of alcoholic HF.

### 4.8. YJSB Downregulated the Contents of α‐SMA, COL‐I, and COL‐Ⅲ in Liver Tissue Homogenates

**Figure 6:**
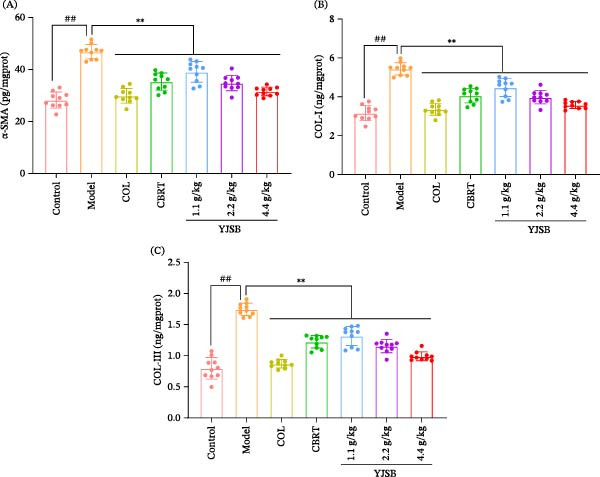
YJSB ameliorated the excessive deposition of extracellular matrix (ECM) in rats (*n* = 10). Levels of α‐SMA (A), COL‐I (B), and COL‐Ⅲ (C) in rat liver homogenates. Comparison with the control group, ^##^ *p* < 0.01. Comparison with the model group,  ^∗∗^ *p* < 0.01.

The contents of α‐SMA, COL‐I, and COL‐Ⅲ in rat liver homogenates were measured to determine if YJSB could mitigate excessive ECM deposition. These levels were significantly elevated in the model group compared to the control group (*p* < 0.01) (Figure 6A–C). Conversely, the levels in the 1.1, 2.2, and 4.4 g/kg YJSB‐treated groups were significantly lower than those in the model group (*p* < 0.01) (Figure 6A–C). These findings suggest that YJSB can mitigate excessive ECM deposition and protect liver function.

### 4.9. YJSB Alleviated the Infiltration of the Liver by Inflammatory Cells in Alcoholic HF Rats

**Figure 7:**
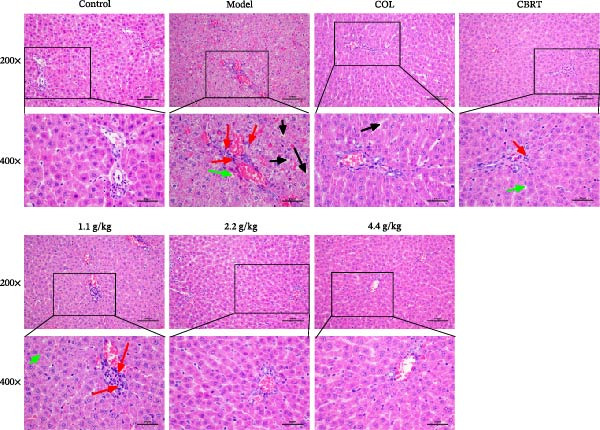
YJSB alleviated liver infiltration by inflammatory cells in rats with alcoholic HF. H&E staining (200× and 400×) (*n* = 6). Red arrow: inflammatory infiltration; black arrow: hydropic degeneration of a few hepatocytes in the liver lobules; green arrow: vesicular steatosis.

H&E staining was used to evaluate the effects of YJSB on improving liver inflammation and structural function in rats with alcoholic HF. In the control group, the liver structure was normal, and no inflammatory cell infiltration was observed. In contrast, the model group exhibited severe hepatocyte necrosis, hydropic degeneration, cystic fatty degeneration, enlarged fusion zones, and inflammatory cell infiltration. Compared with the model group, the liver of rats in the YJSB treatment group showed mild inflammatory cell infiltration, reduced edema, decreased hepatocyte necrosis, reduced hepatic lobule hyperplasia, and improved liver structure (Figure 7). These results indicate that YJSB protects the liver by reducing inflammation and improving the liver structure.

### 4.10. YJSB Repressed the Deposition of Collagen Fibers in the Liver of Alcoholic HF Rats

**Figure 8:**
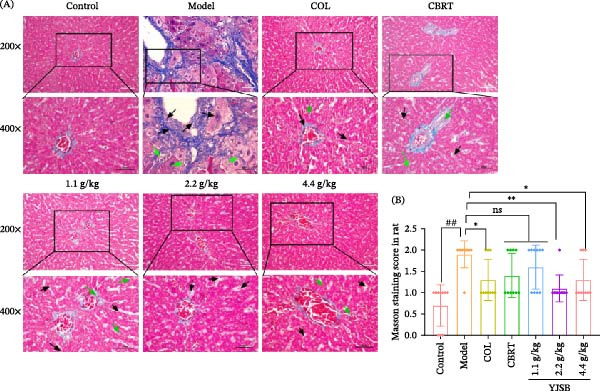
YJSB repressed the deposition of collagen fibers in the livers of rats with alcoholic HF. (A) Masson staining (200× and 400×) (*n* = 6). (B) Rat liver fibrosis score. In comparison with the control group, ^#^ *p* > 0.05, ^##^ *p* < 0.01. Comparison with the model group, ∗^∗^ *p* < 0.01; ns: *p* > 0.05. Black arrows: collagen fibrosis in the confluent area. Green arrows: perisinusoidal collagen fibrosis in the confluent area.

Collagen fibers were detected by Masson staining, with fibrosis staging defined as follows: 0, no collagen fibrosis; 1, fibrosis in the confluent area; 2, fibrosis in the perisinusoidal area of the confluent area; 3, fibrosis in the septum; and 4, cirrhosis [19]. In this study, collagen fibers appeared blue. The model group showed significantly more collagen fiber deposition compared to the control group (*p* < 0.01) (Figure 8A,B). This deposition decreased in the liver tissues of the 1.1 g/kg YJSB‐treated rats (*p* > 0.05) and was significantly lower in the 2.2 and 4.4 g/kg YJSB‐treated groups compared to the model group (*p* < 0.01) (Figure 8A,B). The results indicate that YJSB can effectively inhibit collagen fiber deposition in the liver.

### 4.11. YJSB Attenuated the Deposition of Reticular Fibers in the Livers of Alcoholic HF Rats

**Figure 9:**
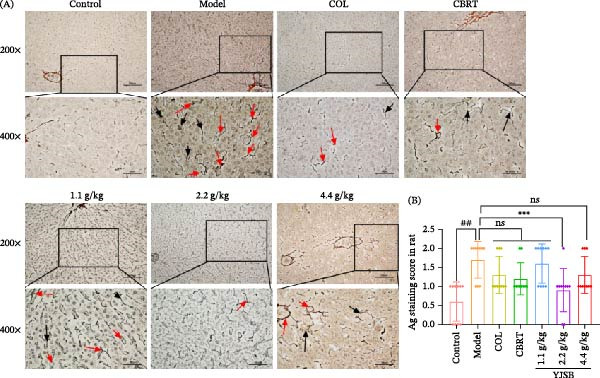
YJSB reduced the deposition of reticulofibrils in the livers of rats with alcoholic HF. (A) Ag staining (200× and 400×) (*n* = 6). (B) Ag staining score of rat livers. Comparison with the control group, ^##^ *p* < 0.01. Comparison with the model group,  ^∗∗^ *p* < 0.05 and  ^∗∗^ *p* < 0.01; ns: *p*  > 0.05. Black arrow: reticulofibrosis in the confluent area; red arrow: perisinusoidal reticulofibrosis in the confluent area.

The deposition of reticular fibers was observed using Ag staining. Fibrosis staging was defined as follows: 0, no fibrosis; 1, reticulofibrosis in the confluent area; 2, perisinusoidal reticulofibrosis in the confluent area; 3, septal reticulofibrosis; and 4, cirrhosis [19]. In the present study, reticular fibers appeared black against a tanned background. The model group exhibited significantly more reticular fiber deposition compared to the control group (*p*  < 0.01) (Figure 9A,B). However, this deposition decreased in the 1.1 g/kg YJSB‐treated group (*p* > 0.05) and was significantly reduced in the 2.2 and 4.4 g/kg YJSB‐treated groups compared to the model group (*p*  < 0.01) (Figure 9A,B). These results indicate that YJSB alleviates the further progression of alcoholic HF by reducing reticular fiber deposition in the liver.

### 4.12. DEG Results

**Figure 10:**
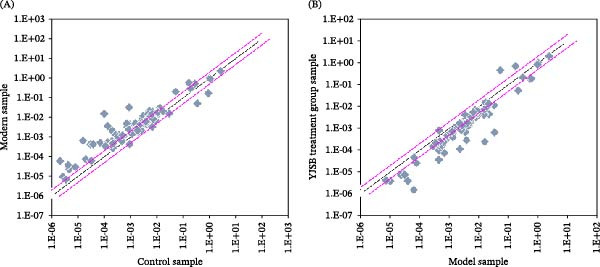
Scatter plot of relative gene expression levels. Black diagonal line: the ratio of relative expression levels between the two groups is 1; points outside the purple diagonal line: the ratio of relative expression levels between the two groups is greater than two‐fold. (A) Relative expression levels of 72 genes between the control group and the model group (*n* = 3) (*X*‐axis: control group; *Y*‐axis: model group). (B) Relative expression levels of 72 genes between the YJSB group and the model group (*n* = 3) (*X*‐axis: model group; *Y*‐axis: YJSB group).

Before performing real‐time PCR amplification, the A260/A280 ratio of the total RNA in the sample was determined by UV absorption and found to be within the range of 1.8 to 2.0, indicating that the total RNA purity was acceptable. Subsequently, the extracted RNA was analyzed by 1% agarose gel electrophoresis. The electrophoresis bands were clear, and the brightness ratio of the two bands of 18S and 28S was ~2:1. The results indicated that all samples were qualified. Functional classification of gene chip results showed that the relative expression ratios of 78 genes in the blank group, model group, and drug administration group differed by more than two‐fold in most cases, indicating significant gene expression differences (Figure 10). We analyzed the expression levels of 72 genes associated with liver fibrosis and found that, compared with the blank group, 65 genes, including MCP‐1, IL‐6, and TGF‐β1, were upregulated in the model group, while seven genes, including CYP2E1, CAT, and GCLM, were downregulated. Among these, the relative expression levels of 31 genes were statistically significant (FC ≥ 2 and *p*  < 0.05) (Table 3). Compared with the model group, 68 genes, including MCP‐1, ATF3, IL‐6, and TGF‐β1, were downregulated in the YJSB treatment group, while four genes, including CYP2E1, CAT, and GCLM, were upregulated. Among these, 34 genes showed statistically significant relative expression levels (FC ≥ 2 and *p*  < 0.05) (Table 3).

**Table 3:** Expression levels of DEG in the model and control groups, YJSB group, and model rat liver tissues (*n* = 3).

Gene name	Model vs. control		YJSB vs. model	
	Fold change	*p*‐Value	Fold change	*p*‐Value
TGF‐β1	**4.08**	**0.0039**	**−** **3.49**	**0.0016**
CTGF	**4.65**	**0.0433**	**−** **3.39**	**0.0247**
LTβR	1.24	0.1708	−1.58	0.0076
MMP‐2	**4.22**	**0.0007**	**−** **2.15**	**0.0336**
PI3K	**2.08**	**0.0026**	**−** **2.19**	**0.0013**
PKC	**4.80**	**0.0049**	**−** **3.20**	**0.0214**
PIK3CD	**3.05**	**0.0155**	**−** **2.03**	**0.0223**
MAPK1	1.40	0.1043	−1.68	0.0061
EP300	1.23	0.1837	−1.44	0.0356
TNF‐α	14.46	0.0614	−4.47	0.0579
IL‐1	−1.15	0.4487	−1.25	0.3464
IL‐6	29.45	0.0525	**−** **41.26**	**0.0214**
NF‐κB	**2.63**	**0.0012**	**−** **2.94**	**0.0003**
Keap1	1.60	0.0269	−1.64	0.0167
Maf	1.04	0.8852	−1.36	0.1027
GCLM	−2.09	0.1460	1.80	0.2581
CYP2E1	−6.68	0.0809	**8.65**	**0.0054**
CREB	1.59	0.0877	**−** **2.13**	**0.0216**
CAT	**−** **5.32**	**0.0053**	**4.09**	**0.0105**
UGT1A6	3.87	0.0890	−2.26	0.1197
Bach1	1.37	0.1303	**−** **3.12**	**0.0014**
p53	**3.11**	**0.0328**	**−** **3.56**	**0.0102**
IKK‐α	1.61	0.0481	−1.62	0.0359
IKKβ	**2.28**	**0.0001**	**−** **2.26**	**0.0003**
P65	**6.45**	**0.0025**	**−** **3.37**	**0.0127**
ATF3	**37.5**	**0.0219**	**−** **51.57**	**0.0073**
iNOS	42.06	0.1554	−9.00	0.1096
HIF‐1a	1.96	0.0530	**−** **2.37**	**0.0138**
CAMK2B	3.66	0.1867	−2.59	0.1718
FLT1	1.25	0.1126	−1.92	0.0044
PFKFB3	**11.19**	**0.0002**	−20.04	1.4273
SERPINE1	**27.43**	**0.0445**	**−** **13.33**	**0.0218**
SLC2A1	3.17	0.0572	**−** **5.13**	**0.0133**
PIK3CB	1.05	0.7863	−1.33	0.1699
PIK3R1	−2.00	0.1284	1.20	0.5894
RELA	1.81	0.0019	**−** **2.49**	**0.0001**
HK2	**7.28**	**0.0077**	**−** **3.90**	**0.0051**
EGFR	−1.84	0.0003	−1.08	0.8756
IGF1R	**2.50**	**0.0104**	**−** **2.43**	**0.0037**
HK1	**3.38**	**0.0028**	**−** **2.54**	**0.0017**
LDHA	1.90	0.2456	−2.92	0.0815
ERBB2	3.80	0.2234	−4.08	0.1111
AKT3	1.82	0.0278	−1.77	0.0227
PLCG2	**3.50**	**0.0005**	**−** **2.13**	**0.0040**
PGK1	**2.44**	**0.0206**	**−** **2.90**	**0.0032**
AKT1	1.78	0.0053	−1.84	0.0026
VEGF	−1.22	0.6977	−1.05	0.6970
GLUT1	1.30	0.5454	−1.85	0.0870
GLUT3	**3.76**	**0.0018**	**−** **3.00**	**0.0012**
IL‐12b	11.42	0.0833	**−** **12.19**	**0.0392**
TLR4	**2.25**	**0.0067**	−1.85	0.0017
MyD88	1.16	0.3915	−1.38	0.0896
IL‐18	**3.29**	**0.0030**	**−** **2.67**	**0.0008**
IRAK1	**2.26**	**0.0203**	**−** **2.10**	**0.0118**
TRAF6	1.33	0.4317	−2.75	0.0702
IL‐1β	6.91	0.0611	−2.16	0.1158
MCP‐1	**154.65**	**0.0108**	**−** **65.42**	**0.0031**
TNFR	1.66	0.0884	−1.94	0.0273
CD40	**4.78**	**0.0031**	**−** **3.37**	**0.0009**
CD27	1.94	0.0803	−1.36	0.3710
CD30	**9.11**	**0.0444**	**−** **10.13**	**0.0187**
BAFF‐R	2.03	0.1480	−1.73	0.2549
RANK	3.24	0.3119	−1.69	0.0729
NIK	**2.18**	**0.0141**	−1.90	0.0119
TRADD	**2.02**	**0.0241**	−1.66	0.0216
RIP1	1.86	0.0194	**−** **2.30**	**0.0036**
TRAF2	**2.83**	**0.0075**	**−** **3.04**	**0.0018**
TRAF5	**3.06**	**0.0007**	−1.65	0.0041
cIAP1	1.62	0.0029	−1.19	0.6810
TAK1	1.54	0.0154	−1.95	0.0059
NEMO	1.17	0.5108	−1.32	0.3058
TAB1	**2.47**	**0.0085**	−1.75	0.0103

*Note:* Data in bold indicate that the corresponding gene is statistically significant (*p* < 0.05). The “−” symbol indicates downregulation of gene expression.

### 4.13. YJSB Attenuated the Release of Inflammation‐Related Factors

**Figure 11:**
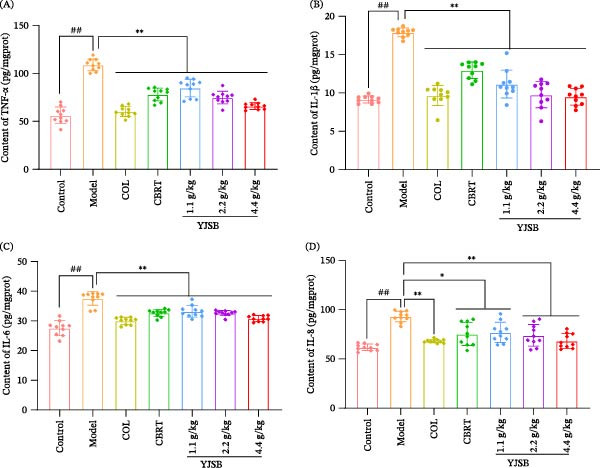
YJSB attenuated the secretion of hepatic inflammation‐related factors (*n* = 10). Levels of TNF‐α (A), IL‐1β (B), IL‐6 (C), and IL‐8 (D) in rat liver homogenates. Comparison with the control group, ^##^ *p* < 0.01. Comparison with the model group,  ^∗^ *p* < 0.05 and  ^∗∗^ *p* < 0.01.

To assess whether YJSB could reduce the release of inflammatory factors, the levels of TNF‐α, IL‐1β, IL‐6, and IL‐8 in rat liver homogenates were measured. The model group exhibited significantly higher levels of these inflammatory markers compared to the control group (*p* < 0.01) (Figure 11A–D). Conversely, the YJSB‐treated groups (1.1 g/kg, 2.2 g/kg, and 4.4 g/kg) showed significantly lower levels of these markers compared to the model group (*p* < 0.05 or *p* < 0.01) (Figure 11A–D). These findings suggest that YJSB mitigates the inflammatory response and protects liver function.

### 4.14. YJSB Improved the Antioxidant Capacity in Alcoholic HF Rats

**Figure 12:**
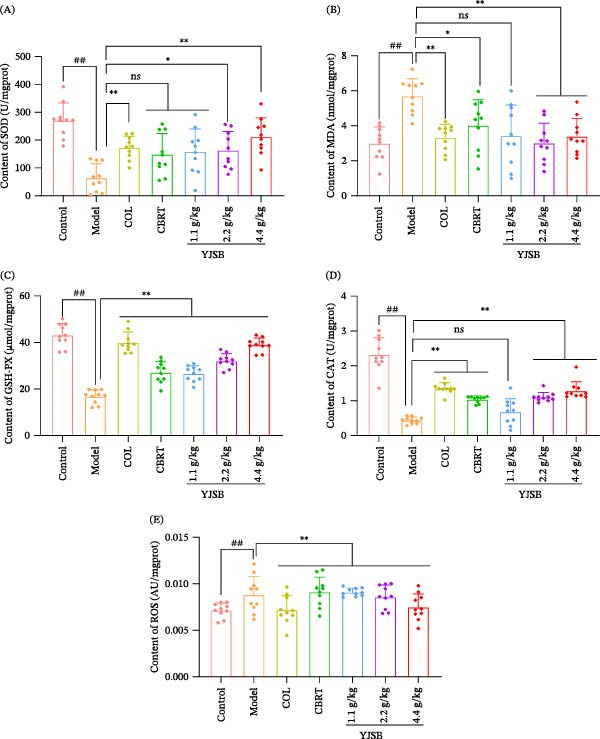
YJSB mitigated the oxidative damage in rats (*n* = 10). Activities or contents of SOD (A), MDA (B), GSH‐PX (C), CAT (D), and ROS (E) in rat liver homogenates. Comparison with the control group, ^##^ *p* < 0.01. Comparison with the model group,  ^∗^ *p* < 0.05 and  ^∗∗^ *p* < 0.01; ns: *p*  > 0.05.

The activities of SOD, GSH‐Px, and CAT were determined, and the levels of MDA and ROS in rat liver homogenates were measured to ascertain the antioxidant effects of YJSB. The model group exhibited significantly higher MDA and ROS levels and significantly lower SOD, GSH‐Px, and CAT activities compared to the control group (*p* < 0.01) (Figure 12A–E). In the 2.2 and 4.4 g/kg YJSB‐treated groups, SOD and CAT activities were significantly elevated, while MDA levels were reduced (*p* < 0.05 or < 0.01) (Figure 12A, B, D). Additionally, GSH‐Px activity was significantly increased, and ROS levels were significantly decreased in the 1.1, 2.2, and 4.4 g/kg YJSB‐treated groups compared to the model group (*p* < 0.01) (Figure 12C,E). In the 1.1 g/kg YJSB‐treated group, SOD and CAT activities increased, though MDA levels did not show significant changes (*p* > 0.05) (Figure 12A, B, D). These results indicate that YJSB has a protective effect on the liver by alleviating oxidative stress damage.

### 4.15. Western Blotting Method for Detecting the Regulatory Effect of YJSB on Proteins in Rat Liver Tissue

**Figure 13:**
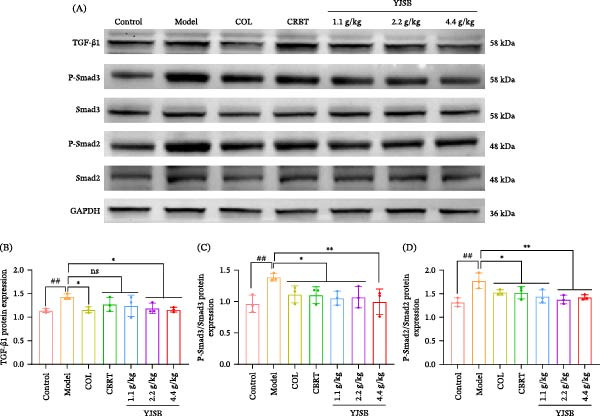
YJSB regulates the protein expression of TGF‐β1, P‐Smad3/Smad3, and P‐Smad2/Smad2 in rat liver tissues (*n* = 3). (A) TGF‐β1, P‐Smad3/Smad3, and P‐Smad2/Smad2 protein blots. (B) Protein expression of TGF‐β1 in rat liver tissues. (C) Protein expression of P‐Smad3/Smad3 in rat liver tissues. (D) Protein expression of P‐Smad2/Smad2 in rat liver tissues. Comparison control group, ^*##*^ *p*  < 0.01. Comparison model group,  ^∗^ *p* < 0.05;  ^∗∗^ *p* < 0.01; ns: *p*  > 0.05.

The mechanism via which YJSB prevents alcoholic HF involves the regulation of Smad pathway proteins. Immunoblotting was used to analyze TGF‐β1, Smad3, P‐Smad3, Smad2, and P‐Smad2 levels in rat livers. These protein levels were upregulated in the model group (*p* < 0.01) (Figure 13A–D) but were significantly downregulated in the 1.1, 2.2, and 4.4 g/kg YJSB‐treated groups compared to the control group (*p* < 0.05 or < 0.01) (Figure 13A‐D).

### 4.16. YJSB Reduced Smad4 Expression in the Nuclei of Hepatocytes From Rats With Alcoholic HF

**Figure 14:**
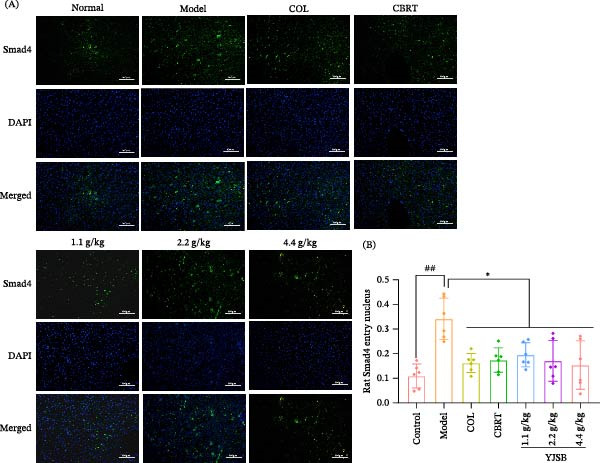
YJSB inhibited the expression of Smad4 in the livers of rats with alcoholic HF. (A) Immunofluorescence map of rat livers (*n* = 6). (B) Statistical analysis of the immunofluorescence densities of the rat liver surfaces. Green: Smad4; blue: 4’,6‐diamidino‐2‐phenylindole (DAPI). Comparison with the control group, ^##^ *p* < 0.01. Comparison with the model group,  ^∗^ *p* < 0.05.

Smad4, a key member of the Smad family and a mediator of the TSSP, plays a significant role in HF pathogenesis [31, 32]. Immunofluorescence was employed to determine the Smad4 levels in the nuclei of rat hepatocytes. Under UV excitation, the nuclei appeared blue, and Smad4 was green. The model group exhibited high Smad4 expression in hepatocyte nuclei (*p* < 0.01) (Figure 14A,B), whereas the 1.1, 2.2, and 4.4 g/kg YJSB‐treated groups showed significantly lower expression compared to the control group (*p* < 0.05) (Figures 14A,B). These findings indicate that YJSB exerts hepatoprotective effects by downregulating the Smad4 expression.

### 4.17. YJSB Can Inhibit the Activation of the TGF‐β1/Smad in HSC‐T6 Cells

**Figure 15:**
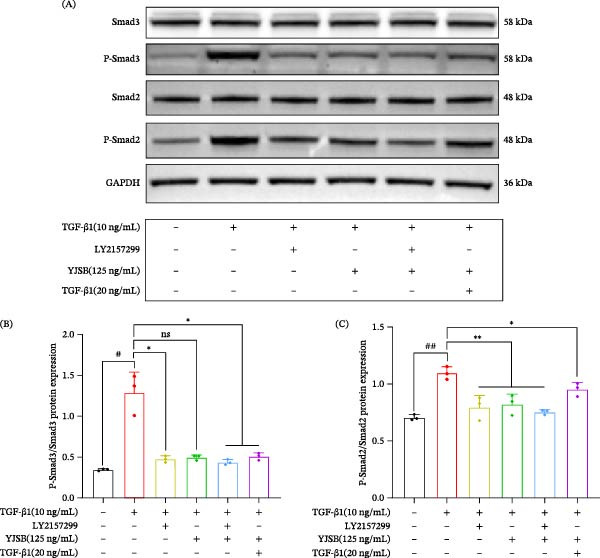
YJSB regulates P‐Smad3/Smad3 and P‐Smad2/Smad2 protein expression in HSC‐T6 cells (*n* = 3). (A) Western blot analysis of P‐Smad3/Smad3 and P‐Smad2/Smad2 proteins. (B) P‐Smad3/Smad3 protein expression. (C) P‐Smad2/Smad2 protein expression. Compared with the control group, ^#^ *p* < 0.05; ^##^ *p* < 0.01. Compared with the model group,  ^∗^ *p* < 0.05;  ^∗∗^ *p* < 0.01; ns: *p*  > 0.05.

Animal studies suggest that YJSB’s mechanism against alcoholic HF may involve the regulation of the TGF‐β1/Smad pathway. To further validate this hypothesis, we conducted in vitro experiments using HSC‐T6 cells. Western blotting results demonstrated that, compared with the blank group, the model group exhibited significant upregulation of P‐Smad3/Smad3 and P‐Smad2/Smad2 proteins (*p* < 0.05 or *p*  < 0.01) (Figure 15A‐C), confirming the hyperactivation of the TGF‐β1/Smad pathway in HSC‐T6 cells. In contrast, significant reductions in P‐Smad3/Smad3 and P‐Smad2/Smad2 were observed in the LY2157299 group (*p* < 0.05 or *p*  < 0.01). The YJSB (125 ng/mL) group showed a significant decrease in P‐Smad2/Smad2 (*p* < 0.01) and a marked downward trend in P‐Smad3/Smad3 (*p* > 0.05). Notably, the combined use of LY2157299 and YJSB exhibited a more pronounced reduction in P‐Smad2/3 protein expression, suggesting a potential synergistic effect in enhancing the inhibition of the TGF‐β1/Smad pathway (*p* < 0.05 or *p*  < 0.01) (Figure 15B,C). Additionally, in the YJSB group, although the inhibitory effect on the TGF‐β1/Smad pathway was partially reversed after exogenous TGF‐β1 (20 ng/mL) stimulation, it was not completely lost. P‐Smad2/3 levels remained significantly lower than those in the model group (*p* < 0.05) (Figure 15B,C). Based on the results of the cell experiments, we conclude that the antifibrotic effect of YJSB is at least partially mediated through the inhibition of the TSSP.

## 5. Discussion

Alcoholic HF is a chronic progressive disease characterized by massive deposition of ECM in the liver, manifested as a process in which fibrous connective tissue gradually replaces liver parenchymal cells, destroying the structure and function of the liver [33]. Liver fibrosis is the core pathological basis for the progression of various chronic liver diseases to cirrhosis and liver cancer [34]. Therefore, exploring how to prevent and treat liver fibrosis in patients with chronic liver disease is of great practical significance. Dai medicine is a medical knowledge system accumulated by the Dai people of China over thousands of years, integrating Indian Ayurveda and traditional Chinese medicine theories [35]. In Dai medicine clinical practice, compound YJSB has been used to treat liver fibrosis and has demonstrated good efficacy and minimal side effects [10].

In modern clinical practice, HF can arise from multiple etiologies, including chronic alcohol abuse, metabolic dysfunction, cholestasis, drug toxicity, viral hepatitis, and autoimmune disorders [36]. Currently, alcohol consumption has become a major risk factor for public health issues such as alcoholic HF, and it is the second leading cause of death worldwide each year [37]. Previous studies have shown that the liver’s metabolism of alcohol produces acetaldehyde, a substance that triggers the liver’s “injury‐repair” process, promotes the transdifferentiation of HSCs into myofibroblasts and drives excessive collagen synthesis and secretion, ultimately leading to worsening liver fibrosis [38, 39]. Therefore, we conducted further validation and in‐depth research on this important HF model of alcoholic HF. In this experiment, a pathway‐focused qPCR array was introduced to screen for DEGs significantly associated with HF, providing insights for more in‐depth and comprehensive mechanism studies.

According to research reports, mice with HF suffer from liver damage resulting in significant elevations in aminotransferases and increased extracellular mesenchymal components in the blood [40]. Transaminases (ALT and AST) are core serum markers used in clinical practice to assess the liver function. Elevated levels indicate liver cell damage or abnormal liver function [41, 42]. Elevated levels of PIIINP, COL‐IV, LN, and HA serum markers reflect the progression of liver fibrosis [43, 44]. Therefore, these indicators were measured in this study to evaluate the liver function and the degree of liver fibrosis in each group of rats. The results showed that YJSB treatment significantly reduced the levels of ALT, AST, PIIINP, COL‐IV, LN, and HA in rat serum, while the opposite was true for the model group. This indicates that YJSB has a protective effect on hepatocytes against alcohol‐induced damage and significantly reduces the deposition of liver ECM, thereby alleviating the progression of alcoholic HF.

Previous studies have shown that TGF‐β1, as a key fibrotic mediator, causes resting HSCs to differentiate into proliferative, migratory, and contractile myofibroblasts, which secrete large amounts of ECM to form scar tissue in the liver [45, 46]. α‑SMA serves as a well‑accepted marker of HSC activation, and its expression level is positively correlated with the degree of fibrosis [47–49]. With the continued activation of HSCs, HSCs secrete large amounts of COL‐I, COL‐III, and Hyp. Hyp, a collagen‐specific amino acid, directly reflects ECM accumulation [50]. The above process leads to pathological ECM overdeposition, forming fibrous septa mainly composed of COL‐I and COL‐III, thereby triggering liver structural remodeling and liver function decline [51]. This experiment found that elevated levels of Hyp, TGF‐β1, α‐SMA, COL‐I, and COL‐III in the model group indicated obvious liver fibrosis lesions in the experimental animals, while administration of YJSB effectively reduced the expression levels of these indicators, indicating that YJSB has excellent efficacy in treating alcoholic HF. It may exert its effects by inhibiting HSC activation and ECM secretion, among other mechanisms.

The alcoholic HF model used in this study recapitulates key morphological and pathophysiological features of human fibrotic liver disease, supporting its clinical relevance and reliability [52]. Histopathological examination confirmed successful model establishment and provided a basis for evaluating the antifibrotic effects of YJSB. In model animals, H&E, Ag, and Masson’s staining revealed marked hepatic inflammation, disorganized hepatocyte architecture, and substantial deposition of collagen and reticular fibers. In contrast, YJSB treatment markedly attenuated these pathological alterations, reducing both inflammatory infiltration and ECM accumulation. These observations underscore the ability of YJSB to suppress HSC activation and mitigate alcohol‑induced fibrotic progression.

Compared with whole‐genome expression profiling chips, pathway‐focused qPCR array uses signal pathway‐oriented designs to detect interacting genes, providing new perspectives for research into disease pathogenesis and drug mechanisms of action and making it more likely to screen for DEGs that have significant biological associations with HF pathogenesis [53]. In this study, we used a pathway‐focused qPCR array to detect the expression of 72 genes highly correlated with HF pathways. According to the quantitative analysis of DEG expression, most genes related to signaling pathways such as Keap1‐Nrf2, TLR4/MyD88, and TGF‐β1 were classified as DEG, indicating that the potential mechanism of action of YJSB against HF is closely related to the regulation of gene expression in pathways such as FGF‐β1, oxidative stress, and inflammatory response. Compared with the model group, TGF‐β1–related DEG (such as TGF‐β1, CTGF, SERPINE1, p53, ATF3, and MMP‐2) were significantly downregulated, with a FC of more than two‐fold, showing a high expression difference, indicating that the TGF‐β1 signaling pathway is the main regulatory pathway of YJSB against alcoholic HF. Therefore, in subsequent experiments, we conducted in‐depth research on the regulatory mechanism centered on the TSSP.

Alcohol, a selective hepatotoxic substance, induces the production of acetaldehyde in the liver, triggering an inflammatory response that prompts HSCs to produce ECM, thereby leading to HF [54]. Inflammation‐related pathways are critically involved in the progression of HF. IL‐6 and IL‐8 promote T‐lymphocyte differentiation and proliferation, further enhancing the inflammatory response [55]. IL‐1β production activates lymphocytes, exacerbating liver damage [56]. TNF‐α promotes hepatocyte apoptosis and exacerbates liver injury by inducing double‐stranded DNA breaks and apoptosis [57, 58]. Elevated levels of inflammatory factors IL‐1β, IL‐6, IL‐8, and TNF‐α directly accelerate HSC activation, contributing to HF progression [59, 60]. This study demonstrated that YJSB administration significantly reduced inflammation‐related molecules in the livers of alcoholic HF‐induced rats. The expression trends of inflammatory factors such as IL‐1β, IL‐6, and TNF‐α were consistent with the results of pathway‐focused qPCR array analysis. This indicates that YJSB inhibits the infiltration of inflammation‐related cells into the liver and alleviates liver fibrosis.

Reduced fibrosis may involve decreased oxidative stress–related factors, such as MDA, and increased SOD activity [61]. The effects of YJSB on these parameters were examined to determine alcohol‐induced hepatocyte injury. The microsomal ethanol oxidation system (MEOS) in the liver metabolizes alcohol, resulting in the overproduction of ROS, including H_2_O_2_ and O^2-^ [61, 62]. These ROS oxidize fatty acids in hepatocyte cell membranes, increasing MDA levels, which disrupt cell membrane and organelle structures, leading to GSH‐Px depletion and extensive hepatocyte damage [62]. SOD is a core antioxidant enzyme that fights oxidative stress by blocking free radical chain reactions and maintaining ROS balance within cells [63]. GSH‐Px serves as an antioxidant barrier, protecting liver cells from ROS damage. MDA serves as an indicator of the extent of oxidative hepatodamage [64]. CAT reduces oxidative stress by breaking down H_2_O_2_ into H_2_O and O_2_ [65]. In this study, YJSB suppressed MDA and ROS levels while enhancing SOD, GSH‐Px, and CAT activities in the livers of alcoholic HF rats. Thus, YJSB elevates the liver’s antioxidant capacity, suppresses oxidative stress–related factors, and reduces oxidative damage to hepatocytes, thereby affecting alcoholic HF.

The onset and progression of HF involve oxidative stress, which exerts its effects through TSSP [66], occurring in the basal and activated states [49, 67]. In the basal state, TGF‐β family ligands are sequestered by the ECM or other binders at the cell membrane, remaining inactive. Upon activation, these ligands are released; TGF‐βI first binds to the TGF‐βⅡ receptor on the cell membrane, recruiting the TGF‐βI receptor to form a TGF‐βI/TGF‐βⅡ receptor complex [19]. The TGF‐βI receptor then undergoes conformational changes, specifically binding to and activating Smad2 and Smad3. The Smad2/3 complex is phosphorylated to P‐Smad2/3 and forms a heterocomplex with Smad4. This heterocomplex translocates into the nucleus, inhibiting genes encoding oxidative stress–related factors. Reduced activities of SOD, GSH‐Px, and CAT and increased levels of MDA and ROS activate HSCs and upregulate α‐SMA, leading to excessive ECM deposition, predominantly COL‐I and COL‐Ⅲ. This process contributes to inflammation, fibrosis, and apoptosis [68]. In this study, the levels of P‐Smad2/Smad2, P‐Smad3/Smad3, and TGF‐β1 were significantly elevated in the livers of rats in the model group, indicating the activation of the TSSP, which finally promoted alcoholic HF progression. However, these proteins were significantly reduced in rats in the YJSB‐treated group, suggesting that YJSB may inhibit HSC activation by suppressing the TSSP, thus ameliorating alcoholic HF. In the TGF‐β1–induced HSC‐T6 liver fibrosis model, Western blotting analysis revealed significantly increased P‐Smad2/3 protein expression in the model group. This finding clearly demonstrates that the TGF‐β1/Smad pathway is in a state of excessive activation within HSC‐T6 cells. Following further intervention with the TGF‐βR1 inhibitor LY2157299, the expression of P‐Smad2/3—the direct product of Smad2/3 phosphorylation upon TGF‐βR1 activation—was significantly reduced. Similarly, the YJSB group exhibited a comparable trend. Notably, the combined use of LY2157299 and YJSB exhibited a more pronounced reduction in P‐Smad2/3 protein expression, suggesting that LY2157299 and YJSB may possess synergistic effects in enhancing the inhibition of the TGF‐β1/Smad pathway. More importantly, following exogenous TGF‐β1 stimulation, the inhibitory effect of the YJSB group on the TGF‐β1/Smad pathway was partially reversed but not completely lost, with P‐Smad2/3 levels remaining lower than those in the model group. This finding provides strong evidence supporting the hypothesis that YJSB may exert its antifibrotic effects in the liver by inhibiting the TGF‐β1/Smad pathway. Similarly, the expression level of Smad4 in the nuclei of liver tissue cells was significantly downregulated in the YJSB‐treated group. This may be attributed to YJSB inhibiting Smad2/3 phosphorylation, reducing the formation of the phosphorylated Smad2/3 + Smad4 complexes, and suppressing the expression of Smad4‐encoding genes. Thus, it increased the levels of oxidative stress factors such as SOD and GSH‐Px, decreased the levels of MDA and ROS, inhibited the activation of HSC, downregulated the expression of α‐SMA, reduced the excessive deposition of the ECM—primarily composed of COL‐I and COL‐Ⅲ—and inhibited the release of inflammatory factors TNF‐α, IL‐1β, IL‐6, and IL‐8. Consequently, it further inhibited the development of fibrosis and apoptosis.

It should be noted that this study employed the liver‐to‐body weight ratio (liver index) as an assessment metric for hepatic indices. Although this is a commonly used measurement method, it exhibits certain limitations in alcoholic HF models. Total body weight may be affected by factors such as ascites, edema, or changes in lean body mass [69]. These fluctuations may affect the accuracy of liver function tests. However, given that both observed histological fibrosis and biochemical markers of injury (such as ALT and AST) showed dose‐dependent improvement under YJSB treatment, we maintain that the core conclusion regarding YJSB’s antifibrotic efficacy remains reliable. In future studies, standardizing liver weight to more stable anatomical parameters (such as tibia length) will enable a more accurate assessment of changes in liver indices.

## 6. Conclusion

In summary, this study revealed that YJSB exhibits antialcoholic HF pharmacodynamic effects, restoring liver function and effectively counteracting alcohol‐induced HF‐related injury. The mechanism of action likely involves inhibiting the inflammatory cascade and mitigating oxidative stress effects by regulating TSSP, thereby hindering alcoholic HF progression.

## Author Contributions

Peixin Guo, Yuhuan Xie, Qinghua Chen, and Linao Zhang designed the research. Linao Zhang and Yuanmei Bai carried out the experiments and performed data analysis. Linao Zhang, Yuanmei Bai, Shifang Luo, Lijie Zheng, Feifan Liu, Xue Wu, and Yan Wan participated in part of the experiments. Linao Zhang wrote the manuscript. Peixin Guo, Linao Zhang, and Yuanmei Bai reviewed and revised the manuscript.

## Funding

This work was supported by the Open Research Fund Program of Yunnan Key Laboratory for Dai and Yi Medicines (Yunnan University of Chinese Medicine) (Grant 2024SS24023, China), the National Natural Science Foundation (Grant 82160867, China), the Yunnan Key Laboratory of Formulated Granules (Grant 202105AG070014, China), and the High‐level Discipline Construction Project of Dai Medicine, National Administration of Traditional Chinese Medicine (Grant zyzdxk‐2023192, China).

## Disclosure

All of the authors have read and approved the final manuscript.

## Ethics Statement

All experimental procedures were performed in accordance with the Guide for the Care and Use of Laboratory Animals and were approved by the Ethics Committee of Yunnan University of Traditional Chinese Medicine (Animal Ethics Number R‐062023033).

## Conflicts of Interest

The authors declare no conflicts of interest.

## Data Availability

The data generated in the present study may be requested from the corresponding author.
